# Transcription Interference and ORF Nature Strongly Affect Promoter Strength in a Reconstituted Metabolic Pathway

**DOI:** 10.3389/fbioe.2015.00021

**Published:** 2015-02-26

**Authors:** Marie Carquet, Denis Pompon, Gilles Truan

**Affiliations:** ^1^Université de Toulouse, INSA, UPS, INP, LISBP, Toulouse, France; ^2^INRA, UMR792 Ingénierie des Systèmes Biologiques et des Procédés, Toulouse, France; ^3^CNRS, UMR5504, Toulouse, France

**Keywords:** metabolic engineering, *Saccharomyces cerevisiae*, combinatorial metabolic libraries, expression tuning, plasmid-encoded metabolic pathway

## Abstract

Fine tuning of individual enzyme expression level is necessary to alleviate metabolic imbalances in synthetic heterologous pathways. A known approach consists of choosing a suitable combination of promoters, based on their characterized strengths in model conditions. We questioned whether each step of a multiple-gene synthetic pathway could be independently tunable at the transcription level. Three open reading frames, coding for enzymes involved in a synthetic pathway, were combinatorially associated to different promoters on an episomal plasmid in *Saccharomyces cerevisiae*. We quantified the mRNA levels of the three genes in each strain of our generated combinatorial metabolic library. Our results evidenced that the ORF nature, position, and orientation induce strong discrepancies between the previously reported promoters’ strengths and the observed ones. We conclude that, in the context of metabolic reconstruction, the strength of usual promoters can be dramatically affected by many factors. Among them, transcriptional interference and ORF nature seem to be predominant.

## Introduction

Many biotechnological processes involve microorganisms to produce molecules of interest in a sustainable way, sometimes competing efficiently with chemical synthesis (Kim et al., 2013). In this context, one of the typical goals of synthetic biology, and more particularly of metabolic engineering, is to genetically modify known and robust microorganisms to create artificial pathways compatible with industrial processes. However, unregulated expression of foreign enzymes used to rebuild synthetic pathways in heterologous hosts may interfere with endogenous metabolic fluxes, causing metabolic burden, overconsumption of resources, or release of toxic intermediates (Glick, 1995; Neubauer et al., 2003). It is thus necessary to properly adjust the levels of all enzymes participating in the heterologous metabolic pathway to maximize metabolic fluxes toward the desired path while avoiding undesirable impacts on cell physiology and viability (Lu and Jeffries, 2007; Ajikumar et al., 2010; Ramon and Smith, 2011). Balancing natural versus synthetic fluxes can be achieved by individually tuning the expression level of each foreign enzyme. In the host *Saccharomyces cerevisiae*, regulated promoters with tunable strengths can be used (Da Silva and Srikrishnan, 2012), but this approach is often limited by the non-linear responses to the inducer concentration or by the cost of these molecules, prohibitive in an industrial context (Siegele and Hu, 1997; Mnaimneh et al., 2004). Alternatively, collections of natural or engineered constitutive promoters of various strengths allow fine tuning of enzyme expression levels (Alper et al., 2005; Lu and Jeffries, 2007; Du et al., 2012).

The importance of promoter strength and regulation on net protein output has been extensively reviewed (Da Silva and Srikrishnan, 2012; Blazeck and Alper, 2013). Some studies characterized constitutive promoters by placing lacZ (Partow et al., 2010) or fluorescent protein encoding ORFs as reporters (Sun et al., 2012; Lee et al., 2013). Lee et al. (2013) characterized a set of *S. cerevisiae* constitutive promoters giving a robust control over gene expression. They used them to build a combinatorial library leading to violacein production without any knowledge of the optimal combination of individual enzymes levels. However, Karim et al. (2013) demonstrated that growth rates and plasmid copy number (parameters known to impact production efficiency) were influenced by additional factors, including the origin of replication and selection marker on episomal plasmids. One can question whether expression levels measured with model systems consistently reflect the strengths of the considered promoters, regardless of their direct genetic environment.

**Figure 1:**
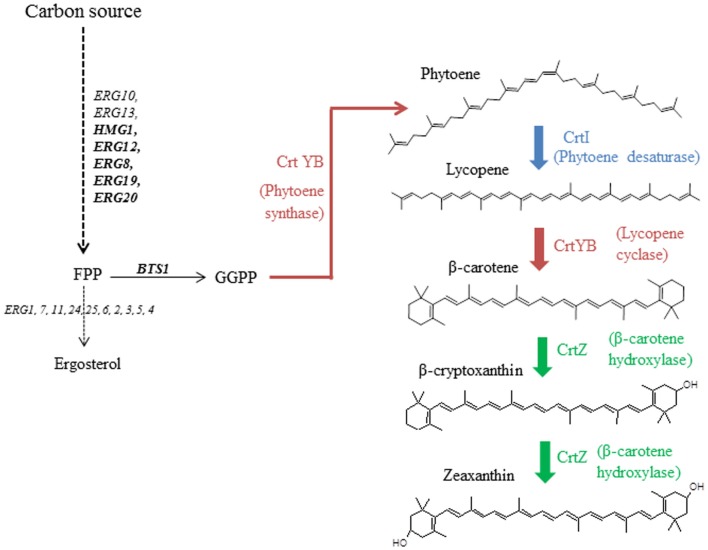
**Zeaxanthin reconstruction pathway in the host *S. cerevisiae* from geranylgeranyl pyrophosphate (GGPP)**. Heterologous steps are colored.

While chromosome integration of genes is the preferred technique for final, industrial strains, first optimization steps are usually performed on plasmids (Ro et al., 2006; Steen et al., 2008; Ukibe et al., 2009; Kocharin et al., 2012). Our goal was to assess whether the assigned strengths of promoters, when described in model systems, can be transposable when multiple expression cassettes are inserted on a single episomal plasmid. We therefore used as model a small, heterologous pathway leading to the synthesis of zeaxanthin, and implemented it in *S. cerevisiae*. The heterologous production of carotenoid molecules has previously been described using *S. cerevisiae* (Verwaal et al., 2007; Ukibe et al., 2009; Sun et al., 2012), demonstrating that rerouting the endogenous terpene pathway to these heterologous metabolites is feasible (Figure 1). Three heterologous ORFs from different carotenoid-producing organisms were used: *CRTI* (phytoene desaturase gene), *CRTYB* (bifunctional phytoene synthase and lycopene cyclase gene), both from *Xanthophyllomyces dendrorhous*, and *CRTZ* (β-carotene hydroxylase gene) from *Pantoea ananatis*. A small metabolic combinatorial library was built using six constitutive promoters that were reported to be strong, medium, or weak using the fluorescent protein GFP as a reporter (Sun et al., 2012). mRNA levels of the three abovementioned genes were quantified in various culture conditions.

## Materials and Methods

### Plasmid constructions

Genes *CRTI* and *CRTYB* from *X. dendrorhous* were amplified from plasmid YEplac195 YB/I (kindly supplied by Prof. Gerhard Sandmann (Verwaal et al., 2007). *CRTZ* from *P. Ananatis* (GenBank accession number D90087) was chemically synthetized (Genecust, Luxembourg) after *S. cerevisiae* codon bias optimization using Gene Designer 2.0 (see Supplementary Material for optimized sequence). *TEF1, PDC1, PGI1, GPD, ENO2*, and *TEF2* promoters, as well as *ADH1, TEF2*, and *CYC1* terminators were amplified from genomic DNA of *S. cerevisiae*. Overlaps of 30 bp were designed between adjacent fragments and no spacer sequences were added between the different transcription units (Table S1 in Supplementary Material). PCR products were purified and further used for overlapping amplification to construct individual expression cassettes (promoter-ORF-terminator). A last PCR amplification was performed with primers RECpRS426_F and RECpRS426_R to extend (40 bp) the recombination segments between the synthetized fragments and pRS426 (Christianson et al., 1992). The library of promoter-ORF-terminator associations was subsequently assembled by Gibson cloning method (Gibson et al., 2009) in a *Sac*I/*Xho*I linearized pRS426 (*URA3* selection marker) (Gibson Assembly Cloning Kit, NEB, Ipswich, MA, USA). The whole construction method is detailed in Figure S1 in Supplementary Material. The combinatorial library is composed of nine plasmids, named pRS426/A to pRS426/I (Table 1). Empty pRS426 is named H. To avoid recombination event, no repeated sequence was inserted into the plasmids. That explains the absence of reuse of the same promoter in each single construct, and the choice of three different terminators. The catalytic subunit of HMG-CoA reductase gene (*tHMG1*) was amplified from genomic DNA of *S*. *cerevisiae*, with primers *tHMG1*_pCM185_F and *tHMG1*_pCM185_R (Table S1 in Supplementary Material) and inserted in pCM185 (TRP1 selection marker) (Gari et al., 1997), resulting in pCM185/*tHMG1*. All constructions were verified by sequencing.

**Table 1:** **Order of genes in the different pRS426 derivatives**.

Constructs	Transcription unit 1			Transcription unit 2			Transcription unit 3		
A	TEF1p	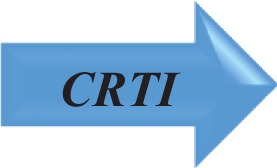	ADH1t	PDC1p	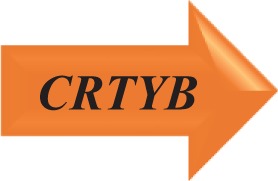	TEF2t	PGI1p	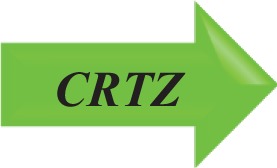	CYC1t
B	TEF1p			PGI1p			PDC1p		
C	PDC1p			TEF1p			PGI1p		
D	PDC1p			PGI1p			TEF1p		
E	PGI1p			TEF1p			PDC1p		
F	PGI1p			PDC1p			TEF1p		
G	GPDp			ENO2p			TEF2p		
H	Empty pRS426									
I	PDC1p	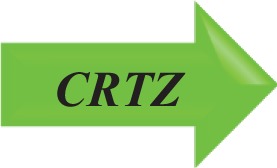	CYC1t	PGI1p	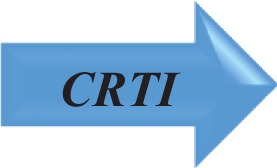	ADH1t	TEF1p	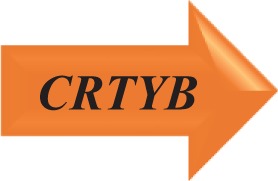	TEF2t
J	PGI1p	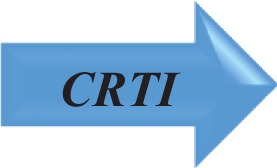	ADH1t	TEF2t	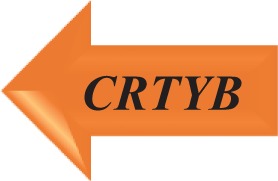	TEF1p	PDC1p	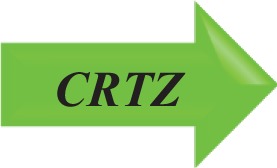	CYC1t

*The strong promoter is shown in pink, medium promoters are in orange, and the weak promoter is in green*.

### Strains, culture, and sampling conditions

The generated vectors were transformed in the *S. cerevisiae* CEN.PK 113-7D strain (*Mata his3 – 1 leu2-3,112 trp1-289 ura3-52 MAL2-8c*) by the lithium acetate method (Gietz et al., 1992), giving birth to strains A to I or A^+H^ to I^+H^, according to the presence of empty pCM185 or pCM185/*tHMG1*, respectively. Recombinant strains were grown on CSM-URA-TRP plates (MP Biomedicals, Santa Ana, CA, USA) from a glycerol stock at 30°C during approximately 3 days. A single colony was then grown in 50 mL of complete synthetic, selective medium (Table S2 in Supplementary Material) at 30°C and 200 rpm during 24 h. 100 mL of the same medium was further inoculated at a final OD_600_ of 0.5, in 500 mL shake flasks. Two milliters of samples were drawn and immediately cooled to 4°C and centrifuged at 3,200 g (4°C). The cell-containing pellets were washed once with 1 mL of ice cold ultrapure water, rapidly centrifuged at 4°C, and quenched in liquid nitrogen before being stored at −80°C for subsequent nucleic acids extraction. At the end of the 72 h of culture, cells were harvested by centrifugation (8,200 g, 2 min), washed once with deionized water, frozen at −80°C, and lyophilized with a CHRIST Alpha 2–4 LD plus freeze-drier. All culture experiments were performed in triplicates. For real independency of triplicates, shake-flasks cultures were performed neither simultaneously, nor from the same inoculum.

### NMR identification and quantification of extracellular compounds

Supernatants were filtered over 0.2 μm and quenched at −80°C prior to their use for NMR quantification of extracellular metabolites on the MetaToul platform. ^1^H-MMR spectra were recorded on a Bruker Avance II 500.13 MHz instrument, equipped with a 5 mm probe BBI. Supernatant samples (500 μL) were mixed with 100 μL of a 4 mM 2-(Trimethylsilyl)propionic-2,2,3,3-d4 acid (TSP-d4) solution in D_2_O as an internal intensity- and chemical shift-standard without further sample pretreatment for metabolite quantification. Measurements were performed with a zgpr pulse program, at 286 K with eight scans per spectrum and a flip angle of 30°(d1: 15 s, TD: 128 K, and presaturation of the ^1^H_2_O signal – O1P: 4,700 ppm). Basic analysis of the acquired spectra and peak integrations were performed using the Bruker “TopSpin 3.0” software suit.

### RT-qPCR experiments

Frozen cells were mechanically disrupted using a Tissue Lyser II (Qiagen, Venlo, the Netherlands) during 3 min at a frequency of 30 Hz. Total nucleic acids were extracted using the GeneJET RNA purification kit (Thermo Scientific, Waltham, MA, USA). Genomic DNA contamination was eliminated by an additional DNase treatment performed with the RNase-free DNase I (Thermo Scientific, Waltham, MA, USA). RNA quality was assayed on a Bioanalyzer 2100 with the RNA 6000 Nano LabChip kit (Agilent Technologies, Santa Clara, CA, USA). One microgram of RNA was reverse-transcribed into cDNA in a 20 μL reaction using the Maxima first strand cDNA synthesis kit (Thermo Scientific, Waltham, MA, USA). Four microliters of a 10-fold dilution of the previously synthetized cDNA were added to the qPCR Master Mix to perform cDNA relative quantification. Quantitative PCR (qPCR) reactions were performed in triplicates, in 20 μL final volume and in 96-well plates (Bio-Rad, Hercules, CA, USA) using the Maxima SYBR Green/Fluorescein qPCR Master Mix (2X) from Thermo Scientific (Waltham, MA, USA), in the MyIQ real-time PCR system from Bio-Rad. A blank (No Template Control) was also incorporated in each assay. Primers were designed using Primer3Plus^1^, and their specificity was verified using the PrimerBLAST tool from NCBI^2^. qPCR primer sequences are given in Table S3 in Supplementary Material. The PCR efficiency of each primer pair(Eff) was evaluated by the dilution series method using a mix of sampled cDNAs as template. The absence of contaminant genomic DNA in RNA preparations was verified using RNA as a template in real-time PCR assays (minus RT control). The thermocycling program consisted of one hold at 95°C for 10 min, followed by 40 cycles of 15 s at 95°C and 60 s at 60°C. After completion of these cycles, melting-curve data were collected to verify PCR specificity, contamination, and absence of primer dimers.

### mRNA variation quantification

Biological samples were prepared in independent triplicates, and qPCR analyses were performed in technical triplicates, bringing to six the number of analysis performed for each tested condition. Four internal control genes were used: *ALG9*, *TFC1*, *UBC6*, and *TAF10*, chosen on their demonstrated expression stability over culture conditions (Teste et al., 2009). All data were calculated using the gene expression module of the BIORAD iQ5 software, using the 2ΔΔCt method (Livak and Schmittgen, 2001). Presented data are average of biological triplicates, normalized to the internal control genes values, with SD. Results were then expressed in terms of mRNA normalized variations, relative to the appropriate reference strain for the observed condition (H or H^+H^). Although these reference strains do not contain the genes *CRTI*, *CRTYB*, and *CRTZ*, some non-specific amplification was detectable in reverse transcription quantitative PCR (RT-qPCR) when the corresponding cDNAs were targeted. Results were normalized by arbitrarily set at 1 the level of signal observed in the reference strains, and expressing mRNA levels in the tested conditions in fold changes relative to the appropriate reference.

### pRS426 copy number determination

Nucleic acids were extracted as described previously. RNA contamination was eliminated by a DNase-free RNase A treatment (Thermo Scientific, Waltham, MA, USA) at 37°C during 10 min, followed by 10 min at 45°C and 10 min at 60°C. Standard curve for plasmid quantification was done as follows: genomic DNA extracted from a native CEN.PK 113-7D strain and three different amounts of pRS426 plasmid were pooled as to mimic 5.3, 10.6, and 15.9 pRS426 copy number per haploid genome. Quantification was performed using *URA3* gene. Twenty nanograms of each mixture was added to the qPCR Master Mix, generating the standard curve. pRS426 quantification in the various strains was performed using the same qPCR amplification starting with 20 ng of extracted DNA (Figure S2 in Supplementary Material).

## Results

### Zeaxanthin pathway library generation

We wanted to understand if promoters of reported strengths (Sun et al., 2012) can be used to impose a precise transcriptional regulation of expression level of genes involved in a synthetic pathway in yeast. Individual ORFs encoding a synthetic zeaxanthin pathway were combinatorially placed under the control of yeast constitutive promoters having different strengths: TEF1p (strong), PGI1p (weak), PDC1p, GPDp, ENO2p, TEF2p (medium), and three independent terminators (see Materials and Methods). An *in vitro* recombination method was used to quickly and reliably obtain the various individual constructs (Figure S1 in Supplementary Material).

The production, in yeast, of mevalonate by HMG-CoA reductase is known to be rate-limiting for the metabolic flux leading to sterols (Donald et al., 1997) and carotenoids (Verwaal et al., 2007; Yan et al., 2012). Therefore, to faithfully reproduce the deregulation strategy, usually applied when producing terpene compounds, and to potentially visualize an effect of precursor boost on heterologous genes expression, a gene encoding the catalytic subunit of HMG-CoA reductase (*tHMG1*) from *S. cerevisiae* was cloned on pCM185 (pCM185/*tHMG1*).

Seven strains were generated: six upon transformation with the six plasmids harboring various promoter strengths and one corresponded to the transformation with the seventh plasmid harboring medium strengths promoters. The reference strain was transformed with the empty pRS426. These eight generated strains (Table 1) were further transformed with pCM185 or pCM185/*tHMG1*, leading to 16 different strains named A to H when transformed with empty pCM185 (named set 1 throughout the rest of this document) or A^+H^ to H^+H^ when transformed with pCM185/*tHMG1* (named set 2 throughout the rest of this document).

### *S. cerevisiae* strains, culture, and sampling

**Figure 2:**
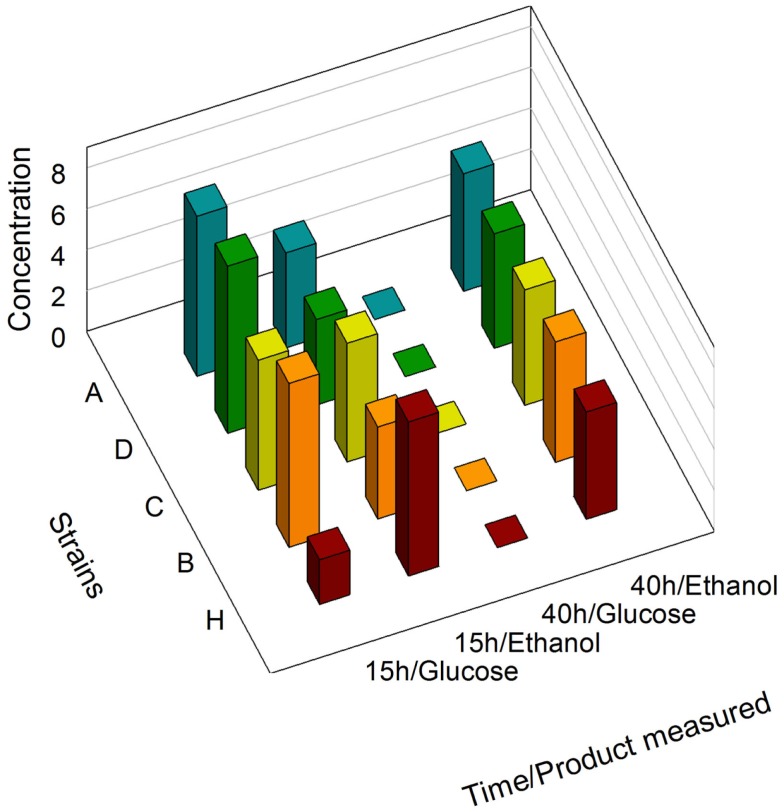
**NMR determination of glucose and ethanol concentrations**. Supernatants of strains A, B, C, D, and H were analyzed after 15 h and 40 h of growth.

We first determined that no significant differences existed between the growth curves of the zeaxanthin producing strains and the reference strains (Figure S3 in Supplementary Material). During batch growth on glucose, yeast cells shift from fermentation to respiration (diauxic shift) after exhausting the primary carbon source, causing extensive reprogramming of the transcription machinery (Galdieri et al., 2010). *GPD*, *PDC1*, *PGI1*, and *ENO2* genes are involved in both glucose and ethanol assimilation processes. Consequently, the associated promoters are potentially sensitive to the cell metabolic state. We analyzed by NMR the supernatant of the culture medium of the strains A, C, D, and H (Figure 2). After 15 h of growth, the medium contained glucose as well as ethanol produced by fermentation. Interestingly, the glucose concentration was slightly lower in strain H than in any other strain harboring the zeaxanthin pathway, although the difference between the growth curves measured for strain H compared to the zeaxanthin strain was within error range. However, it is possible that the reference strain H consumed glucose slightly faster than any of the zeaxanthin producing strains. On the contrary, after 40 h of growth, only ethanol could be found in the medium. We therefore determined that at 15 h of growth, the strains were still fermenting glucose (preferred carbon source); while at 40 h of growth, they had shifted to respiratory conditions (consuming ethanol). mRNA extraction and quantification were then performed either in fermentative context (15 h, glucose) or in respiratory context (40 h, ethanol).

### pRS426 copy number quantification

**Figure 3:**
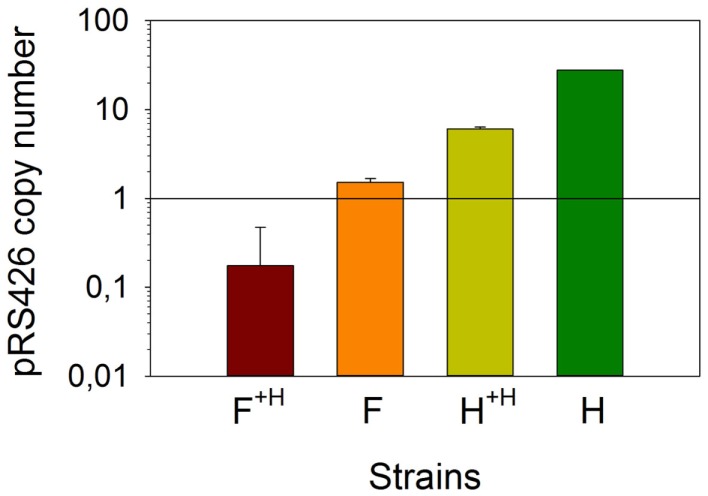
**Absolute quantification of the copy number of pRS426/F**. Strains F and F^+H^ were compared with pRS426 in reference strains H and H^+H^.

Prior to analyze the transcription variation of the combinatorial metabolic library genes, we investigated whether the copy number of the pRS426 derivatives changed in the different strains. We determined that strain H contained approximately 28 ± 0.1 copies of the empty pRS426, a regular value for a 2 μ based plasmid (Romanos et al., 1992) (Figure 3). Using strain F as a representative for all zeaxanthin producing strains, we determined that the pRS426/F copy number was 2.0 ± 0.2 copies per cell only. It has been demonstrated that in case of deleterious gene products, a decrease in the plasmid copy number was observed (Fang et al., 2011). Zeaxanthin biosynthesis may lead to a certain toxicity, resulting in a positive selection pressure on cells harboring a reduced number of the pRS426 derivatives. Interestingly, adding a second, monocopy plasmid (pCM185/*tHMG1*) in these two strains (F and H, generating F^+H^ and H^+H^ respectively) promoted a systematic decrease of the copy number of pRS426 derivatives (6.0 ± 0.3 copies for H^+H^ compared to 28 ± 0.1 for strain H and about 0.5 ± 0.3 copy for F^+H^ compared to 2.0 ± 0.2 copies in strain F). This suggests a possible interference between the two plasmids, as already observed in *Escherichia coli* (Gruber et al., 2008). As a conclusion, the copy number of pRS426 derivatives was affected by the zeaxanthin pathway expression and the presence of pCM185/*tHMG1*.

### Expression profile of the pRS426 marker

**Figure 4:**
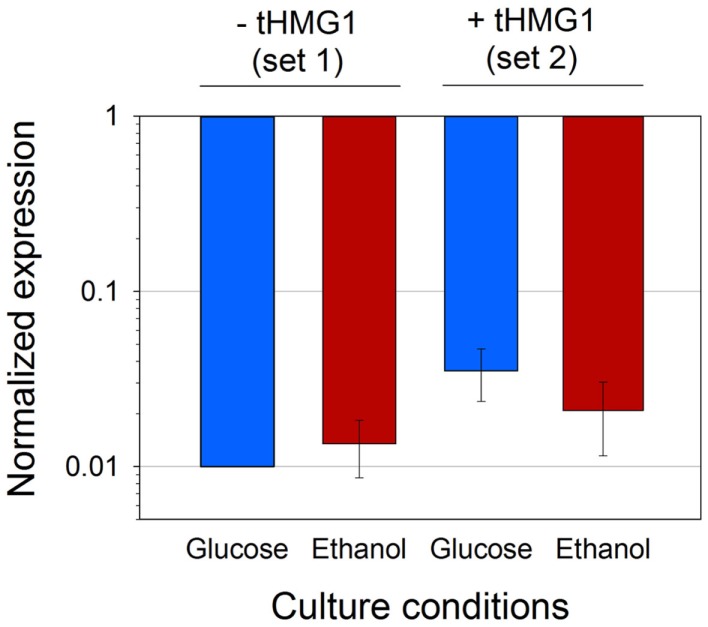
**Average *URA3* mRNA level in four conditions**. Analyses were performed on set 2 or set 1 (with or without t*HMG1* expression, respectively), in glucose (blue bars) or ethanol (red bars) metabolism. Results were normalized with *URA3* signal in strain H for strains from set 1, and with *URA3* signal in strain H^+H^ for strains from set 2. Error bars represent 1 SD.

Given the discrepancies in the copy number of pRS426 derivatives when *tHMG1* or carotenoid genes were expressed, we wanted to confirm if this variation was reflected in mRNA levels of the *URA3* gene present in the pRS426 backbone (Figure S4 in Supplementary Material). mRNA levels of *URA3* in each set of strains (set 1 or set 2) were averaged, and standardized to the levels calculated from the reference strain H or H^+H^, respectively (Figure 4). First, the fairly minor SD showed that the variation of the *URA3* mRNA levels was minimal between the different strains. Secondly, the mean values of mRNA levels were almost constant over the different conditions (small variations being within error range), but dramatically reduced (50–100-folds) in zeaxanthin producing strains, when compared to the reference. As mentioned earlier, the copy number of pRS426 derivatives decreased down to approximately 1 in the strains harboring the zeaxanthin pathway compared to approximately 30 copies in the reference strains. Consequently, the strong decrease in the *URA3* mRNA levels could hardly be explained by the sole reduction of the copy number of the pRS426 derivatives. The three heterologous genes were placed far away and downstream the *URA3* gene in pRS426 (Figure S4 in Supplementary Material), theoretically limiting possible transcriptional effects between them. Nonetheless, transcription of *CRTI*, *CRTYB*, and *CRTZ* may interfere in some way with *URA3* promoter activity either due to some competition between the different promoters or possible changes in the plasmid DNA structure, hampering a correct DNA unwinding prior transcription. A regulation of endogenous *ura3-52* gene present in CEN.PK may also take part in the *URA3* mRNA level reduction. Indeed, the addition of a plasmid containing the *URA3* gene may modify transcriptional regulation of endogenous uracil pathway.

### Controls of plasmid integrity and cell physiology

**Figure 5:**
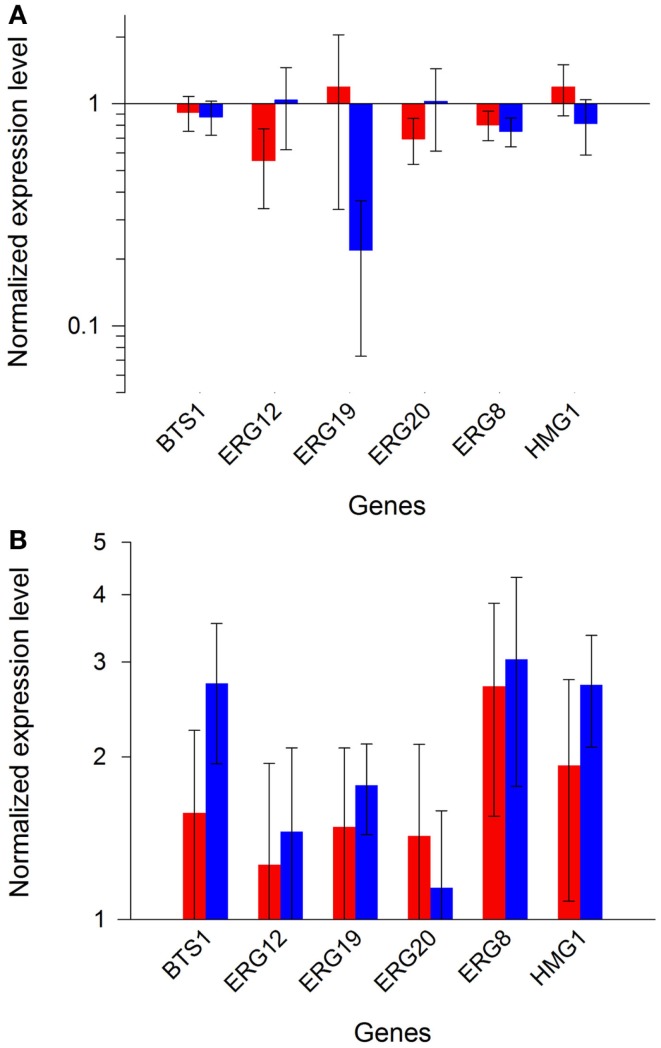
**Expression profiles of endogenous GGPP pathway genes**. Bars represent the mean value of the different gene expressions in the strains from set 1 [**(A)**, red and blue bars for glucose or ethanol metabolism, respectively], or in the strains from set 2 [**(B)**, red and blue bars for glucose or ethanol metabolism, respectively]. Error bars represent 1 SD.

In order to ascertain reproducibility of our quantifications, several controls experiments were conducted. To ensure that no recombination events occurred between one of the plasmids and *S. cerevisiae* genome, or between the two plasmids, we verified their integrity after transformation and cultivation. Restriction analyses (Figure S5 in Supplementary Material), performed on extracted plasmids, demonstrated that the specific bands of pRS426 and pCM185 derivatives corresponded to normal restriction profiles. We then verified if the expression of the different pRS426 derivatives could lead to changes in the transcriptional regulation of some specific genes. mRNA levels of a restricted number of genes involved in the production of endogenous carotenoids precursors were evaluated in the 14 engineered strains. *BTS1*, *ERG20*, *ERG19*, *ERG8*, *ERG12*, and *HMG1* were chosen as targets because of their implication in the production of geranylgeranyl pyrophosphate (GGPP), the precursor involved in the first step of the heterologous biosynthetic pathway (Figure 1). For *HMG1* mRNA quantification, primers were designed to avoid detection of transcripts from *tHMG1*. There was almost no impact of the carotenoid pathway on the transcriptional response of the six chosen genes when *tHMG1* was not expressed (level changes are within the error range) (Figure 5A). However, when *tHMG1* was expressed, there was a slight tendency to overexpress the GGPP pathway genes (Figure 5B). However, this increase is small compared to the technical error on this experimental design. In any case, the effects were minor and equivalent between all producing strains (no more than 3× the reference level) compared to non-producing strains H and H^+H^. Therefore, the production of zeaxanthin in our conditions did probably not strongly impact the pool of precursors and no physiological adaptation was therefore seen at the level of GGPP pathway regulation.

### Absence of correlation between promoters strengths and mRNA levels

**Figure 6:**
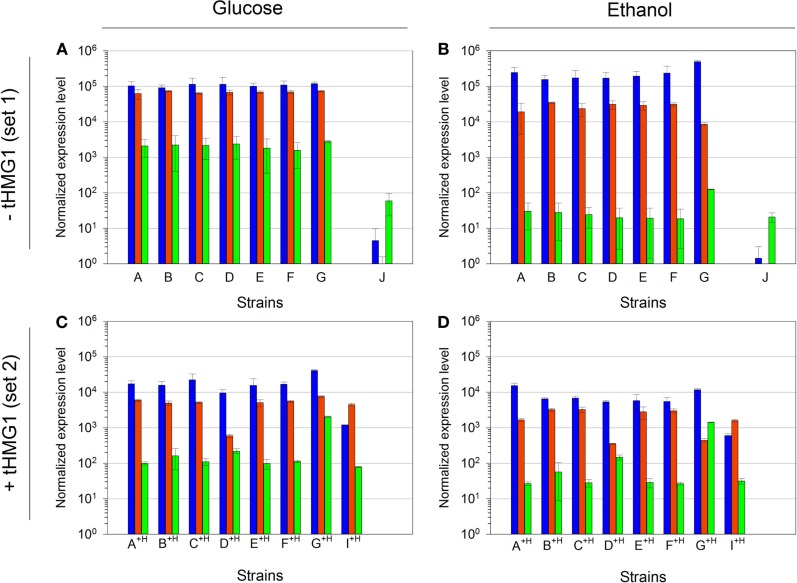
**Quantitative RT-PCR analysis of mRNA levels of the three implemented carotenoid genes**. **(A)** mRNA levels in strains A to J, in glucose metabolism (normalized with strain H); **(B)** mRNA levels in strains A to J, in ethanol metabolism (normalized with strain H); **(C)** mRNA levels in strains A^+H^ to I^+H^, in glucose metabolism (normalized with strain H^+H^); **(D)** mRNA levels in strains A^+H^ to I^+H^, in ethanol metabolism (normalized with strain H^+H^). Bar colors are as follows: *CRTI*, blue; *CRTYB*, red; and *CRTZ*, green. Error bars represent 1 SD. mRNA levels for strain J in glucose metabolism were done in duplicate and the error bars represent the two maximum values.

We analyzed the normalized mRNA levels of *CRTI*, *CRTYB*, and *CRTZ* genes for each strain and in every growth context. We again emphasize that only the promoter nature (and strength) in front of each ORF is different in strains from set 1 or set 2, independently (Table 1). Surprisingly, Figure 6 highlights one major profile in these sets of strains that can be described as follows: *CRTI* is the most expressed gene, followed by *CRTYB* and finally by *CRTZ*. The general tendency shows lower levels of mRNAs in set 2 (in accordance with the pRS426 derivatives copy number decrease when *tHMG1* was expressed) or in respiratory conditions. However, in strain G^+H^, *CRTZ* mRNA level (in glucose) was not impacted by *tHMG1* and even augmented compared to the one measured in strain G. Furthermore, in strain G^+H^, *CRTZ* mRNA level was not modified between glucose and ethanol conditions, unlike *CRTI* and *CRTYB* mRNA levels. In construct G, TEF2p, controlling *CRTZ* transcription, is the only promoter not to be involved in glucose or ethanol assimilation. This may be related to the *CRTZ* mRNA level stability over culture conditions. This results in *CRTYB*, *CRTI*, and *CRTZ* mRNA levels variation less than 15-fold in strain G^+H^, a feature that is in good accordance with the expected mRNA levels imposed by the chosen promoters (medium strength promoters for all three genes) (Sun et al., 2012). In conclusion, in our experimental conditions, mRNA levels did not reflect the previously described strengths of promoters (Sun et al., 2012).

### Gene orientation and order strongly affect promoters strengths

We tested the hypothesis that the mRNA levels in our system were dependent on the genes’ position. We designed two new plasmids, keeping the promoter-ORF-terminator associations of pRS426/E, but swapping the positions of the three genes (pRS426/I) or reversing the *CRTYB* transcription unit orientation (pRS426/J) (Table 1). Due to very poor transformation efficiency of CEN.PK with these new plasmids, only strains I^+H^ and J were generated.

Compared to strain E^+H^, *CRTI* mRNA level in strain I^+H^ was decreased by a factor of 10 while the mRNA levels of *CRTYB* and *CRTZ* were constant (Figures 6C,D). This phenomenon was not dependent on culture conditions, as it was true in glucose and in ethanol metabolisms. A failure of *CRTYB* transcription termination may explain the observed diminution of *CRTI* mRNA level. Although TEF2t (terminator associated to *CRTYB*) was not tested in Curran et al. (2013) study, its efficiency may not be sufficient to promote the normal release of the entire transcription machinery at the termination site. This phenomenon may hinder the RNA polymerase fixation at *CRTI* promoter. Therefore, changing the *CRTI* transcription unit position could alter the promoter strength, demonstrating that the construction structure and more precisely the relative gene position interfered with its expression level.

Relative quantifications of mRNA levels in strain J pointed a strong decrease of *CRTI* and *CRTYB* mRNAs (Figures 6A,B). In this strain, *CRTYB* mRNA was absent and *CRTI* mRNA level was strongly decreased, while the *CRTZ* mRNA level was lowered only by a factor of 10. To ascertain that no recombination events could have modified the plasmid harboring the zeaxanthin pathway, we verified (restriction and sequence analysis) that all three genes were still present and correctly ordered on the pRS426/J extracted from strain J (Figure S5 in Supplementary Material). In pRS426/J, *CRTI* and *CRTYB* were in a convergent configuration, potentially leading to a collision between the two opposite RNA polymerases. This phenomenon may render difficult their progress toward the terminator. Furthermore, during the transcriptional process, the RNA polymerases progress on DNA making positive supercoils on both strands simultaneously (Liu and Wang, 1987). This may create a region of hyper-supercoiling that could possibly prevent further advancement in either direction on the template, leading to the suppression of the transcriptional processes of *CRTI* and *CRTYB*. Hence, in our experiments, *CRTI* mRNA level seemed to be affected by the gene position and orientation while *CRTYB* mRNA level was impacted only by the gene orientation.

## Discussion

Our series of experiments reveal that the promoter-independent control of mRNA levels may result from several different mechanisms. The ORF nature, *via* the sequence related variation of mRNA half-life, may strongly influence the mRNA level. Occurrence of cryptic promoters in ORFs (Pedersen et al., 1999) might also promote or inhibit transcription independently of the promoter-induced regulation. Gene terminators are also known to influence mRNA stabilization (Curran et al., 2013; Yamanishi et al., 2013). Curran et al. (2013) reported that the terminator of *CYC1* gene (CYC1t) was a poor expression enhancing terminator. Therefore, the use of CYC1t behind *CRTZ* may explain the low levels of its corresponding mRNA. Overall, the quasi absence of correlation between the strengths of the different promoters and their corresponding mRNA levels strongly suggests that the relative ORF positions on the plasmid may drive transcription efficiency. Experiments performed with the I and J strains reveal that *CRTYB* ORF nature also imposed a regulation of its transcription, and that the observed suppression of *CRTI* and *CRTYB* transcription in strain J was probably due to poor terminator (TEF2t) efficiency. As *CRTZ* mRNA level was the most stable over the different tested conditions, its ORF nature or associated terminator (CYC1t), or both, seemed to impose a strong transcriptional regulation.

Parts of the effects seen in these two last constructions might also be attributed to transcription interference (TI). TI is defined as the suppressive influence of one transcriptional process, directly and in cis, on a second transcriptional process (Shearwin et al., 2005). This phenomenon was well-studied with native *S. cerevisiae* genes (Prescott and Proudfoot, 2002; Wang et al., 2014) but only two studies addressed the case of TI in heterologous expression, demonstrating transcription inhibition effects only in the case of divergent genes (Bae et al., 2008; Lee et al., 2014). It is known that the genome of *S. cerevisiae* is highly compact (an ORF every 2 kb in average) and less than 6% of the convergent adjacent genes show a pattern of co-expression. This suggests that a counter-selection pressure for co-transcribed convergent genes exists (Dujon, 1996). Prescott and Proudfoot (2002) described a drastic effect on the transcription processes of two convergent genes when intergenic sequences containing genes terminators were removed. They interpreted this phenomenon by a collision, in the absence of terminators, between RNA polymerases simultaneously transcribing the two genes. These effects were abolished when the two transcriptional processes were not simultaneous, confirming the strong TI in the case of simultaneous transcription. Contrarily to previous studies (Bae et al., 2008; Lee et al., 2014), our results provide a clear indication that relative positioning of three contiguous transcription units and their orientations probably induce TI on a multicopy plasmid. Large TI effects demonstrated in our design may be related to terminators efficiency, and are probably accentuated by the absence of extra-space between our three transcription units.

We show here that the strengths of chosen promoters, as given by the available literature data, are not systematically conserved when used for the reconstruction of foreign pathways. TI, ORF nature, and possibly terminator may also counterbalance the initially imposed regulation. In strains from set 1, we observed a factor of 1,000 between *CRTI* and *CRTZ* mRNA levels, a surprising result considering that the expected regulation of mRNA levels by promoters strengths is supposedly a factor of 10 between the strongest and the weakest promoters (Sun et al., 2012). Overall, we propose that combinatorial metabolic libraries [similar to COMPACTER method (Du et al., 2012)], associating various promoters/terminators to heterologous genes and varying their positions is probably a quick and reliable method to generate diversity at the level of transcription regulation. However, due to possible unexpected transcriptional effects, we propose that a systematic analysis of all heterologous genes mRNA levels should be conducted to link the desired metabolite production level to the real pattern of transcription. This may allow researchers to select the best combination according to the production of the desired molecule.

## Author Contributions

MC designed the study, made experiments, and wrote this manuscript; GT and DP designed the study and wrote this manuscript.

## Conflict of Interest Statement

The authors declare that the research was conducted in the absence of any commercial or financial relationships that could be construed as a potential conflict of interest.

## Supplementary Material

The Supplementary Material for this article can be found online at http://www.frontiersin.org/Journal/10.3389/fbioe.2015.00021/abstract

## Footnotes

^1^http://www.bioinformatics.nl/cgi-bin/primer3plus/primer3plus.cgi/

^2^http://www.ncbi.nlm.nih.gov/tools/primer-blast/

## Abbreviations

GGPP, geranylgeranyl pyrophosphate; GFP, green fluorescent protein; RT-qPCR, reverse transcription quantitative PCR; qPCR, quantitative PCR; tHMG1, truncated HMG-CoA reductase.
