# Supplementary material for: Transcription Interference and ORF Nature Strongly Affect Promoter Strength in a Reconstituted Metabolic Pathway
Source: Front Bioeng Biotechnol. 2015 Feb 26;3:21. doi: 10.3389/fbioe.2015.00021 (PMC4341558; doi:10.3389/fbioe.2015.00021)
Supplement: Supplementary file 1 [file datasheet_1.pdf]

Supplementary Material

Transcription interference and gene nature strongly affect promoter strength in a reconstituted metabolic pathway.

Marie Carquet[1,2,3], Denis Pompon[1,2,3], Gilles Truan[1,2,3]*

[1]Université de Toulouse; INSA, UPS, INP; LISBP, Toulouse, France
[2]INRA, UMR792 Ingénierie des Systèmes Biologiques et des Procédés, Toulouse, France
[3]CNRS, UMR5504, Toulouse, France*

*** Correspondence:** Gilles Truan, [1]Université de Toulouse; INSA, UPS, INP; LISBP, 135 Avenue de Rangueil, F-31077 Toulouse, France
gilles.truan@insa-toulouse.fr

1. Supplementary Data

1.1 Sequence optimization

CRTZ ORF sequence optimized according to *Saccharomyces cerevisiae* codon bias:

```
1       ATGTTATGGA TCTGGAACGC TTTGATCGTT TTCGTTACTG TTATCGGTAT GGAAGTTATC
61      GCTGCTTTGG CCCACAAGTA CATTATGCAC GGTTGGGGTT GGGGTTGGCA CTTATCCCAC
121     CACGAACCAC GTAAGGGTGC CTTCGAAGTT AACGACTTGT ACGCCGTTGT TTTCGCCGCT
181     TTGAGCATCT TGTTGATTTA CTTGGGTAGC ACCGGTATGT GGCCATTACA ATGGATCGGT
241     GCTGGTATGA CCGCCTACGG TTTATTGTAC TTCATGGTTC ACGACGGTTT AGTTCACCAA
301     CGTTGGCCAT TCCGTTACAT TCCACGTAAG GGTTACTTAA AGCGTTTGTA CATGGCCCAC
361     AGAATGCACC ACGCCGTTCG TGGTAAGGAA GGTTGTGTTA GTTTCGGTTT CTTGTACGCC
421     CCACCATTAT CCAAGTTGCA AGCTACTTTG AGAGAACGTC ACGGTGCTAG AGCCGGTGCC
481     GCCAGAGATG CTCAAGGTGG TGAAGATGAA CCAGCCAGCG GTAAGTGA
```

1.2 Carotenoid quantification

Carotenoid extraction and assay. Authentic astaxanthin (Cayman Chemical, Ann Arbor, MI) was added as an internal standard (10 µL of 1mM astaxanthin for 400 mg of dried cells). 2 g of 0.5 mm diameter beads were added to the freeze-dried cells. The pellets were vortexed at maximum speed during 2 minutes to separate dried cells. The suspension was then vortexed three times in 5 mL of acetone during 2 minutes with chilling cycles on ice in between. Cells were further incubated in acetone, on ice and in the dark during 3 hours. Cell debris and glass beads were separated from the supernatant by centrifugation at 8,200g for 2 min at 4°C. Pellets were washed twice with 5mL of acetone and supernatants were pooled. Extracts were evaporated to dryness under nitrogen flux and resuspended in 1 mL of isopropanol. A last centrifugation step was performed at 1,150g and at 4°C during 2 min to clarify the extracts before HPLC injection. 10 µL of sample were loaded onto the Agilent ZORBAX SB-C18 column and analyzed using a Waters 2690 Separation Module. The HPLC program was set as a flow rate of 1 mL/min: 0–3 min, 50% (v,v) H_2O-25% isopropanol-25% acetonitrile (MeCN); 3–20 min, linear gradient from 50% H_2O-25% isopropanol-25% MeCN to 10% H_2O-70% isopropanol-20% MeCN; 20–25 min,10% H_2O-70% isopropanol-20% MeCN; 25–35 min, linear gradient from10% H_2O-70% isopropanol-20% MeCN to 50% H_2O-25% isopropanol-25% MeCN. Column temperature was set at 60°C. Authentic zeaxanthin and lycopene from Extrasynthèse France (Lyon), and β-carotene from Sigma-Aldrich (St-Louis, MO) were used as standards and monitored by HPLC at 450 nm. Standard curves were performed by dilution series of these standards for quantification purposes.

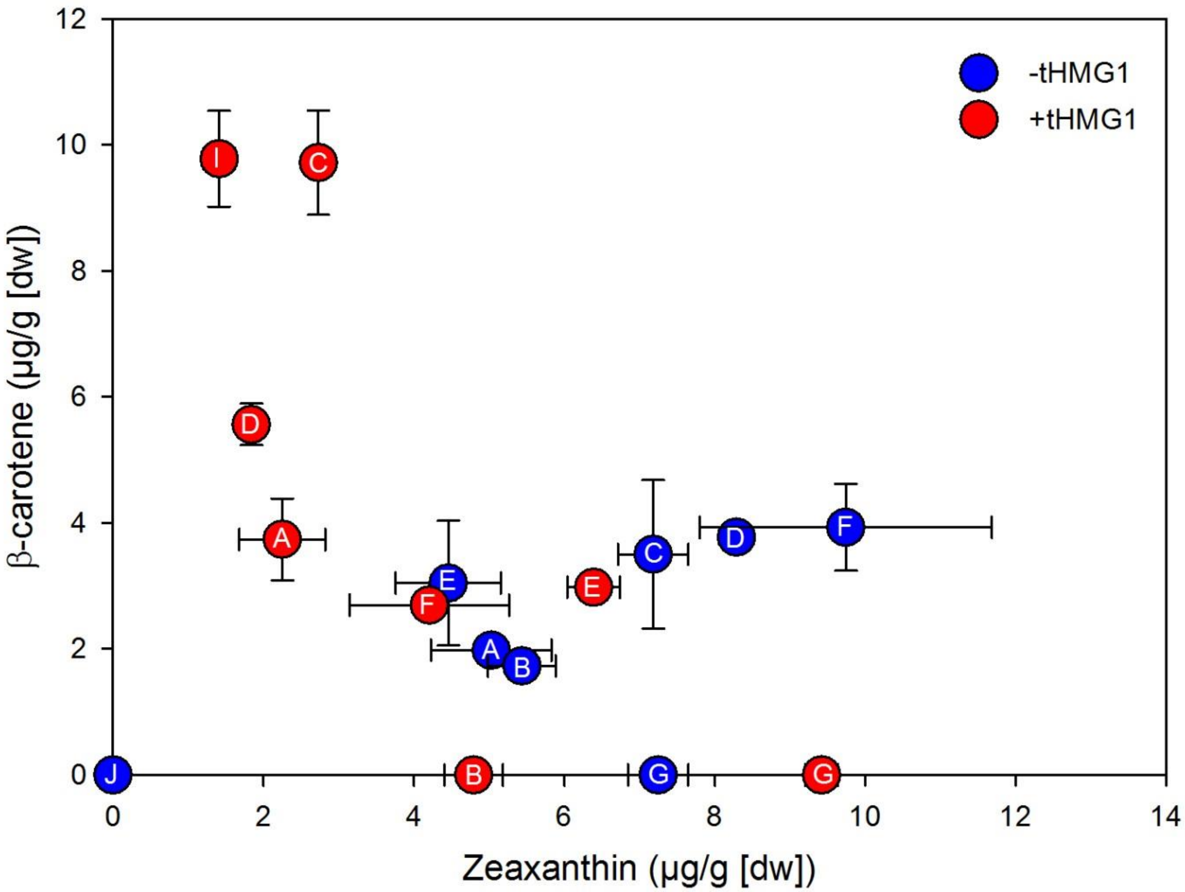

Absence of correlation between zeaxanthin and β-carotene productions in strains A^{+H} to G^{+H}. Strains expressing *tHMG1* are shown in red, others are in blue.

Carotenoid production discussion. We quantified carotenoid production of strains $A^{(+H)}$ to $G^{(+H)}$ in stationary phase condition (72 hours). The figure depicted above evidences two distinct groups: in absence of *tHMG1* expression, strains generally produce more zeaxanthin than β-carotene (blue points group), and in presence of *tHMG1* expression, strains generally produce less zeaxanthin but more β-carotene (red points). In our conditions, *tHMG1* expression seams to boost β-carotene rather than zeaxanthin synthesis. Synthesis of zeaxanthin from β-carotene is catalyzed by CrtZ. To our knowledge, no biochemical data is available about CrtZ activity or stability in yeast. We hypothesize that the additional flux of precursor induced by *tHMG1* expression may be consumed by CrtI and CrtYB but not by the low levels of CrtZ, resulting in β-carotene accumulation in strains $A^{(+H)}$ to $F^{(+H)}$. However, one strain design (strain G) was performant for zeaxanthin production, without any β-carotene accumulation. In this case only, we observed an improvement in zeaxanthin production due to *tHMG1* expression. We previously demonstrated that strain G^{+H} was unique in that *CRTZ* gene presented an mRNA level equivalent to *CRTI* and *CRTYB* at the end of the culture (Fig. 5D). We conclude that this metabolic pathway requires approximately equivalent expression levels of *CRTI*, *CRTYB* and *CRTZ* genes to efficiently produce zeaxanthin. *tHMG1* expression is a good strategy to produce carotenoid derivatives, but may not be systematically relevant if any gene in the pathway is expressed at low levels, resulting in the accumulation of precursors immediately before the considered reaction.

2. Supplementary Figures and tables

2.1. Supplementary Tables

Supplementary Table 1. Primer sequences used to assemble the varying promoter zeaxanthin pathways.

Target	final construct	Primer name[a)]	Primer sequence (5'-> 3')[b)]
CRTI	TEF1p_CRTI	pRS426_TEF1p_F	ATTGGGTACCGGGCCCCCCCATAGCTTCAAAATGTTTCTA
		TEF1p_CRTI_R	TTGTTCTTTTCCCATTTTGTAATTAAAACTTAGAT
		CRTI_TEF1p_F	AGTTTTAATTACAAAATGGGAAAAGAACAAGATCA
	PDC1p_CRTI	pRS426_PDC1p_F	ATTGGGTACCGGGCCCCCCCATGCGACTGGGTGAGCATA
		PDC1p_CRTI_R	TTGTTCTTTTCCCATTTTGATTGATTTGACTGTGT
		CRTI_PDC1p_F	GTCAAATCAATCAAAATGGGAAAAGAACAAGATCA
	PGI1p_CRTI	pRS426_PGI1p_F	ATTGGGTACCGGGCCCCCCCTAACAAAAATCACGATCTGG
		PGI1p_CRTI_R	TTGTTCTTTTCCCATTTTTAGGCTGGTATCTTGAT
		CRTI_PGI1p_F	GATACCAGCCTAAAAATGGGAAAAGAACAAGATCA
	GPDp_CRTI	pRS426_GPDp_F	ATTGGGTACCGGGCCCCCCCAGTTTATCATTATCAATACT
		GPDp_CRTI_R	TTGTTCTTTTCCCATATCCGTCGAAACTAAGTTCT
		CRTI_GPDp_F	TTAGTTTCGACGGATATGGGAAAAGAACAAGATCA
	CRTI_ADH1t	CRTI_ADH1t_R	CATAAGAAATTCGCTTCAGAAAGCAAGAACACCAA
		ADH1t_CRTI_F	GTTCTTGCTTTCTGAAGCGAATTTCTTATGATTTA
	Primers designed to overlap with next cassette	ADH1t_TEF1p_R	ACATTTTGAAGCTATGGCATGCCGGTAGAGGTGTG
		ADH1t_PDC1p_R	TCACCCAGTCGCATGGGCATGCCGGTAGAGGTGTG
		ADH1t_PGI1p_R	TCGTGATTTTTGTTAGGCATGCCGGTAGAGGTGTG
		ADH1t_ENO2_R	CCGCAGCGTCGACACGGCATGCCGGTAGAGGTGTG
CRTYB	TEF1p_CRTYB	TEF1p_ADH1t_F	CTCTACCGGCATGCCATAGCTTCAAAATGTTTCTA
		TEF1p_CRTYB_R	TGCGAGAGCCGTCATTTTGTAATTAAAACTTAGAT
		CRTYB_TEF1p_F	AGTTTTAATTACAAAATGACGGCTCTCGCATATTA
	PDC1p_CRTYB	PDC1p_ADH1t_F	CTCTACCGGCATGCCCATGCGACTGGGTGAGCATA
		PDC1p_CRTYB_R	TGCGAGAGCCGTCATTTTGATTGATTTGACTGTGT
		CRTYB_PDC1p_F	GTCAAATCAATCAAAATGACGGCTCTCGCATATTA
	PGI1p_CRTYB	PGI1p_ADH1t_F	CTCTACCGGCATGCCTAACAAAAATCACGATCTGG
		PGI1p_CRTYB_R	TGCGAGAGCCGTCATTTTTAGGCTGGTATCTTGAT
		CRTYB_PGI1p_F	GATACCAGCCTAAAAATGACGGCTCTCGCATATTA
	ENO2p_CRTYB	ENO2p_ADH1t_F	CTCTACCGGCATGCCGTGTCGACGCTGCGGGTATA
		ENO2p_CRTYB_R	TGCGAGAGCCGTCATTATTATTGTATGTTATAGTA
		CRTYB_ENO2p_F	TAACATACAATAATAATGACGGCTCTCGCATATTA
	CRTYB_TEF2t	CRTYB_TEF2t_R	CAATAATTATTACTCTTACTGCCCTTCCCATCCGC
		TEF2t_CRTYB_F	TGGGAAGGGCAGTAAGAGTAATAATTATTGCTTCC
	Primers designed to ovelap with next cassette	TEF2t_TEF1p_R	ACATTTTGAAGCTATGATGAGGCCGTCTTTTGTTG
		TEF2t_PDC1p_R	TCACCCAGTCGCATGGATGAGGCCGTCTTTTGTTG
		TEF2t_PGI1p_R	TCGTGATTTTTGTTAGATGAGGCCGTCTTTTGTTG
		TEF2t_TEF2p_R	TAAGTATACGGCCCCGATGAGGCCGTCTTTTGTTG

Target	final construct	Primer name[a)]	Primer sequence (5'-> 3')[b)]
CRTZ	TEF1p_CRTZ	TEF1p_TEF2t_F	AAAGACGGCCTCATCATAGCTTCAAAATGTTTCTA
		TEF1p_CRTZ_R	CCAGATCCATAACATTTTGTAATTAAAACTTAGAT
		CRTZ_TEF1p_F	AGTTTTAATTACAAAATGTTATGGATCTGGAACGC
	PDC1p_CRTZ	PDC1p_TEF2t_F	AAAGACGGCCTCATCCATGCGACTGGGTGAGCATA
		PDC1p_CRTZ_R	CCAGATCCATAACATTTTGATTGATTTGACTGTGT
		CRTZ_PDC1p_F	GTCAAATCAATCAAAATGTTATGGATCTGGAACGC
	PGI1p_CRTZ	PGI1p_TEF2t_F	AAAGACGGCCTCATCTAACAAAAATCACGATCTGG
		PGI1p_CRTZ_R	CCAGATCCATAACATTTTTAGGCTGGTATCTTGAT
		CRTZ_PGI1p_F	GATACCAGCCTAAAAATGTTATGGATCTGGAACGC
	TEF2p_CRTZ	TEF2p_TEF2t_F	AAAGACGGCCTCATCGGGGCCGTATACTTACATAT
		TEF2p_CRTZ_R	CCAGATCCATAACATGTTTAGTTAATTATAGTTCG
		CRTZ_TEF2p_F	TATAATTAACTAAACATGTTATGGATCTGGAACGC
	CRTZ_CYC1t	CRTZ_CYC11t_R	CATGATGCGGCCCTCTCACTTACCGCTGGCTGGTT
		CYC1t_CRTZ_F	GCCAGCGGTAAGTGAGAGGGCCGCATCATGTAATT
		CYC1t_pRS426_R	CTAAAGGGAACAAAAGCTGGGGCCGCAAATTAAAGCCTTC
		RECpRS426_F	TACGACTCACTATAGGGCGAATTGGGTACCGGGCCCCCCC
		RECpRS426_R	AAGCGCGCAATTAACCCTCACTAAAGGGAACAAAAGCTGG
tHMG1	pCM185/*tHMG1*	*tHMG1*_pCM185_F	ATTACCGGATCAATTCGGGGATGGACCAATTGGTGAAAACTGAAG
		*tHMG1*_pCM185_R	CAGGCCTGTTTAAACGGATCTTAGGATTTAATGCAGGTGACGGAC

a) Primer names are constructed as follow: target sequence_overlapping sequence_F/R as it is a Forward or a Reverse primer.

b) Overlapping sequences are shown in underlined letters.

Supplementary Table 2: Culture medium composition.

**Salts**	H3PO4 85%	0.66%
	KH2PO4	21 mM
	MgSO4, 7H2O	2.4 mM
	MnSO4, H2O	94.7 µM
	CuSO4, 5H2O	1.60 µM
	ZnSO4, 7H2O	52.2 µM
	CoCl2, 6H2O	11.8 µM
	Na2MoO4, 2H2O	10.3 µM
	H3BO3	121.4 µM
	Citric acid	2.60 µM
	KI	6 µM
	NiSO4, 6H2O	15.4 µM
	Trisodium citrate, 2H2O	85 µM
**Vitamins**	Thiamine HCl	6 µM
	Pyridoxine HCl	24.3 µM
	nicotinic acid	65 µM
	D-Biotin	0.20 µM
	Ca-D-Panthothenate	21 µM
	Myo-inositol	444.2 µM
**Other components**	CaCl2, 2H2O	448 µM
	FeCl3, 7H2O	173.4 µM
	Glucose	20 g/L
	Casaminoacids	10 g/L
	Leucine	0.01% final
	Histidine	0.01% final

Supplementary Table 3: Primers used for RT-qPCR analyses.

Target	forward Primer 5' > 3'	Reverse Primer 5' > 3'
URA3	AACAGTATAGAACCGTGGATGATGT	CTGTAACGTTCACCCTCTACCTTAG
CRTI	GTTCACGTCCTGCAGAAGAACT	CTCTTGTCCAGATAGACTCGAAGG
CRTYB	CGAACTCCTTAGAGGATACACCAC	GAGCCTGCTACACATAGACCATAG
CRTZ	TACTTGGGTAGCACCGGTATGT	GGTGAACTAAACCGTCGTGAAC
BTS1	ATAGGGGACAAGGCTTGGAT	GAGACAGCGCTTCCATGAGT
ERG20	ACACTCCAGGCGGTAAGCTA	CCTGCAACAACTCAATGCAC
ERG19	CATGCCACATGTTTGGACTC	CAACACAGCATTTGGACCTG
ERG8	TCGAACCTCCCGTACAAACT	TAGCGGTTTGAGCCCTAAGA
ERG12	TGGACTGCTTGTCTCAATCG	AGCACCGGTAAGTTTTGTGG
HMG1[(a)]	AGACCCGTTTGACGTCCTTA	GTAGAGGCGCTCAACCAAAA

(a) The HMG1 primer couple targets the 5’ HMG-CoA reductase sequence, before the truncation giving birth to tHMG1 occurs. Consequently it targets only the endogenous copy of HMG-CoA reductase mRNA.

2.2. Supplementary Figures

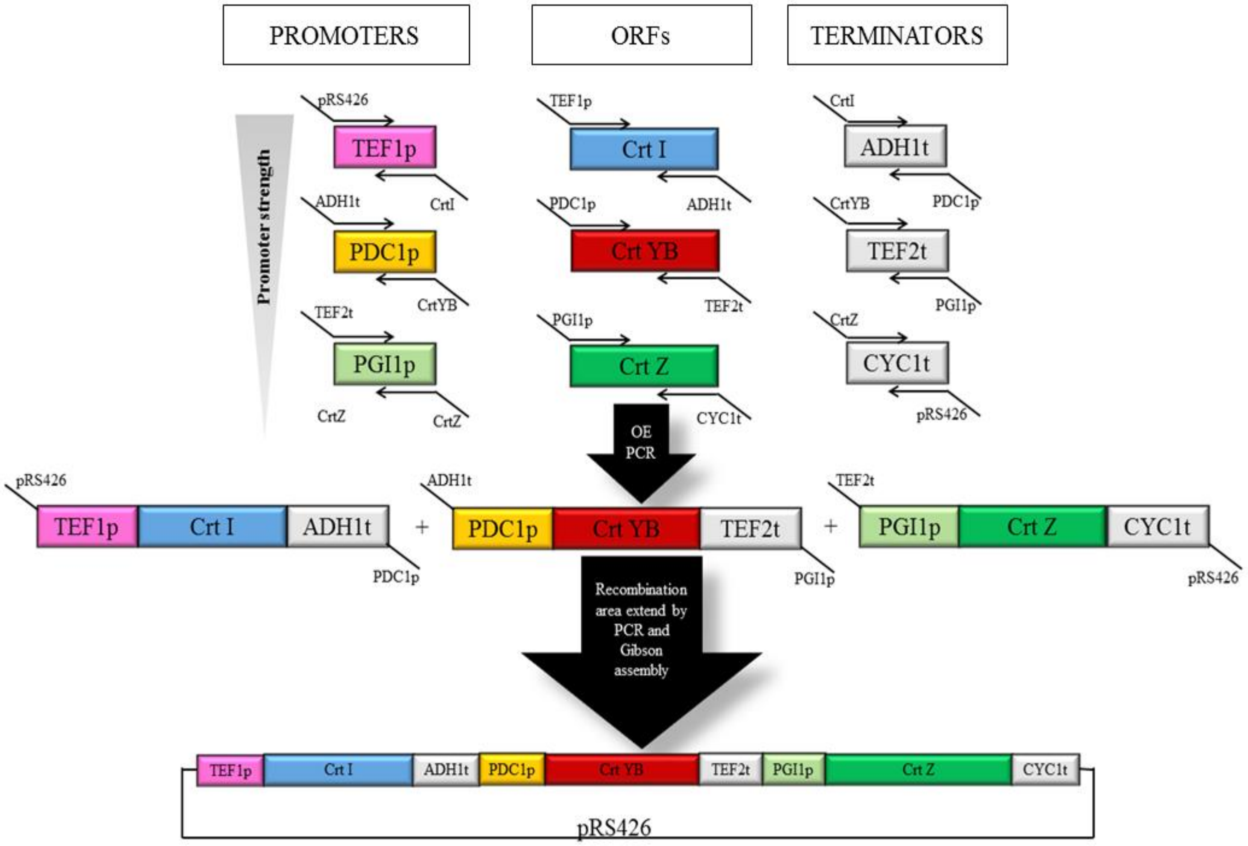

Supplementary Figure 1: Example of construct pRS426/A. Construction method: each element (promoters, genes, and terminators) was PCR amplified with overlapping ends in the primers shown here with arrows. OE PCR permitted to obtain the different expression cassettes. In a single step, three of them were then assembled by Gibson method (Gibson et al., 2009) in a vector pRS426. Strong promoter is shown in pink, medium promoters are in orange and weak one is in green. The example of construct A is shown here.

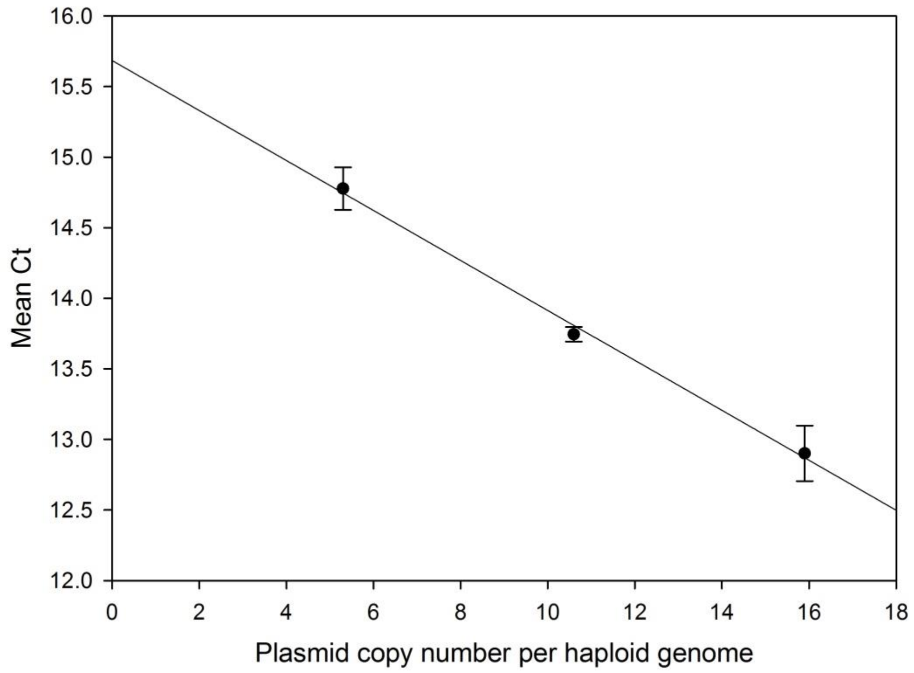

Supplementary Figure 2: Standard curve for absolute quantification of pRS426 derivatives copy number. Known quantities of pRS426 were added to CEN.PK 113-7D DNA extract, mimicking 5.3, 10.6 and 15.9 copies of pRS426. Each experiment was done in triplicate. Error bars correspond to one standard deviation. We represent here mean Ct values obtained for three different pRS426 copy number per haploid genome. Small Ct values correspond to large amounts of pRS426, according to the qPCR method in which a large amount of DNA give rise to an early amplification signal.

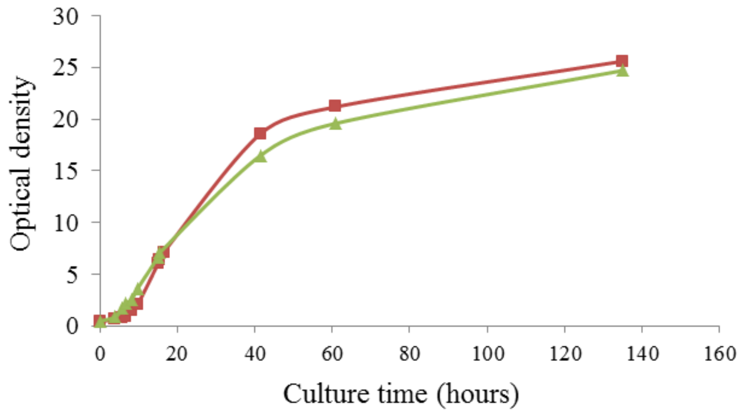

Supplementary Figure 3: Growth parameters of the zeaxanthin producing strains. Few strains are taken here for example, but every strain behaved the same.
Growth curves followed by optical density at 600 nm, with either the zeaxanthin producing strain C^{+H} (red line, squares) or with the reference strain H (green line, triangles).

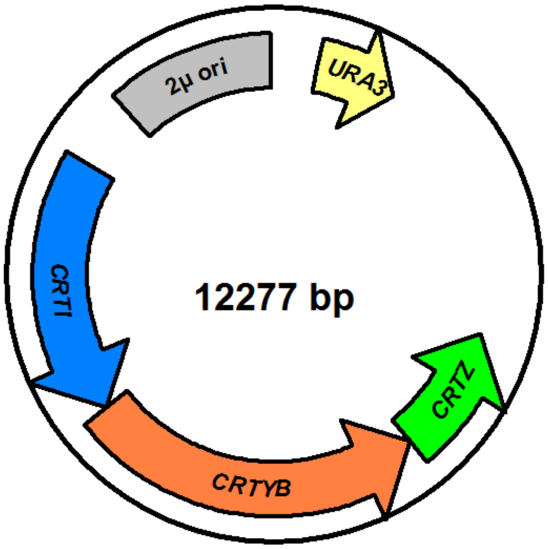

Supplementary Figure 4: pRS426 derivative map. Example of construct A is shown here.
2µ ori: replication origin 2µ. *URA3*: selection marker.

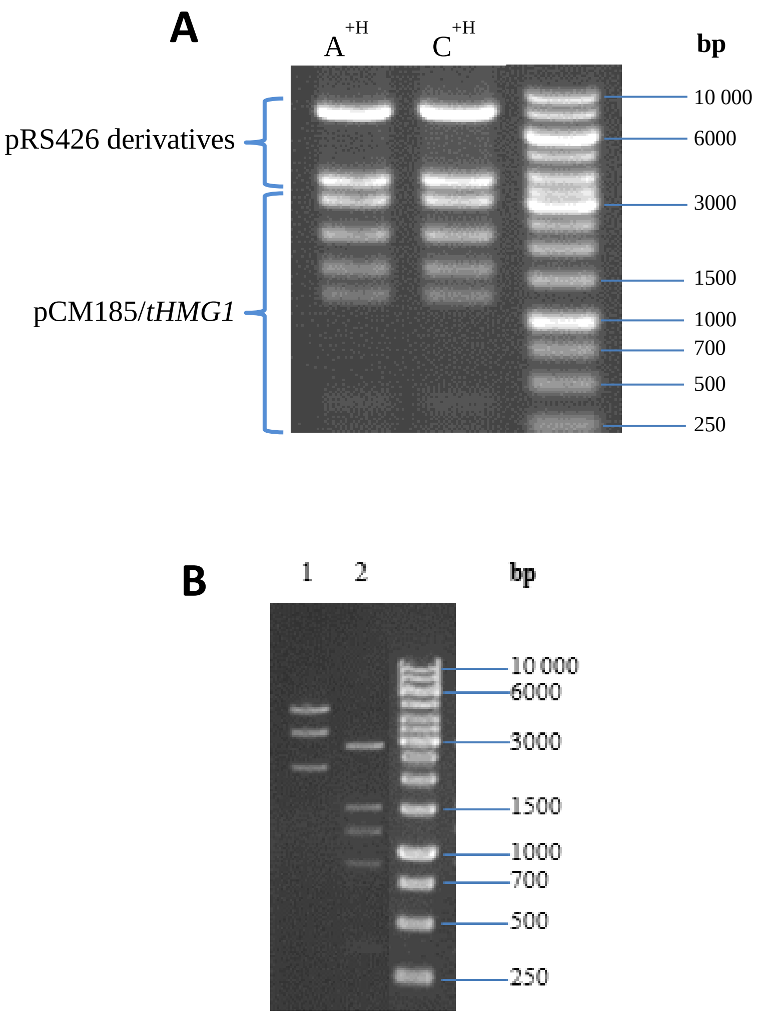

Supplementary Figure 5: Restriction profiles of plasmids recovered from zeaxanthin producing strains and *BglI* digested. Migration on 0.8% agarose gel.

(**A**) Plasmids recovered from strains A^{+H} and C^{+H} are shown here as examples, but result was the same in every zeaxanthin producing strain. Expected bands for the pRS426 derivatives are 8245 bp and 4032 bp. Expected bands for plasmid pCM185/*tHMG1* are 3286 bp; 2301 bp; 1646 bp; 1305 bp; 371 bp; 122 bp and 71 bp. (**B**) Plasmids recovered from strain J. 1 = pRS426/J ; 2 = pCM185. Expected bands for pRS426/J are : 5701 bp ; 4032 bp et 2544 bp. Expected bands for pCM185 are : 3286 bp ; 1646 bp ; 1305 bp ; 929 bp ; 371 bp ; 122 bp et 71 bp.

DNA bands were BET stained and revealed under UV light. We cannot see the smallest bands in this gel because of insufficient amounts of DNA, but visible bands correspond to expected profiles.

3. References

Gibson, D.G., Young, L., Chuang, R.Y., Venter, J.C., Hutchison, C.A., 3rd, and Smith, H.O. (2009). Enzymatic assembly of DNA molecules up to several hundred kilobases. *Nat Methods* 6**,** 343-345. doi: 10.1038/nmeth.1318.
